# Hospital-based surveillance of enteric parasites in Kolkata

**DOI:** 10.1186/1756-0500-2-110

**Published:** 2009-06-19

**Authors:** Avik Kumar Mukherjee, Punam Chowdhury, Mihir Kumar Bhattacharya, Mrinmoy Ghosh, Krishnan Rajendran, Sandipan Ganguly

**Affiliations:** 1 National Institute of Cholera and Enteric Diseases, P-33, C. I. T. Road, Beliaghata, Scheme XM, Kolkata-700010, West Bengal, India; 2 ID & BG Hospital, Kolkata, India

## Abstract

**Background:**

Diarrhoea is the second leading cause of illness and death in developing countries and the second commonest cause of death due to infectious diseases among children under five in such countries. Parasites, as well as bacterial and viral pathogens, are important causes of diarrhoea. However, parasitic infections are sometimes overlooked, leading after a period of time to an uncertain aetiology. In this paper we report the prevalence of *Giardia lamblia*, *Entamoeba histolytica *and *Cryptosporidium *sp. in and around Kolkata.

**Findings:**

A hospital-based laboratory surveillance study was conducted among the patients admitted between November 2007 and October 2008 to the Infectious Diseases (ID) Hospital (Population = 1103) with diarrhoeal complaints. Of the 1103 samples collected, 147 were positive for *Giardia lamblia*, 84 for *Cryptosporidium *sp. and 51 for *Entamoeba histolytica*. For all these parasites there was a high rate of mixed infection with common enteric viruses and bacteria such as Rotavirus, *Vibrio cholerae *and *Shigella *sp. There were also cases of co-infection with all other diarrheogenic pathogens. The age group ≥ 5 years had the highest prevalence of parasites whereas the age group >5 – 10 years was predominantly infected with *Giardia lamblia *(p =< 0.001; Odds ratio (OR) = 3.937; 95% Confidence interval (CI) = 1.862 – 8.326) and with all parasites (p = 0.040; OR = 2.043; 95% CI = 1.033 – 4.039). The age group >10 – 20 years could also be considered at risk for *G. lamblia *(p = 0.009; OR = 2.231; 95% CI = 1.223 – 4.067). Month-wise occurrence data showed an endemic presence of *G. lamblia *whereas *Cryptosporidium *sp. and *E. histolytica *occurred sporadically. The GIS study revealed that parasites were more prevalent in areas such as Tangra, Tiljala and Rajarhat, which are mainly slum areas. Because most of the population surveyed was in the lower income group, consumption of contaminated water and food could be the major underlying cause of parasitic infestations.

**Conclusion:**

This study provides important information on the occurrence and distribution of three important intestinal parasites and indicates their diarrheogenic capacity in Kolkata and surrounding areas.

## Background

Diarrhoea is a leading cause of illness and death in developing countries. A global estimate suggests that 19% of total child deaths are attributable to diarrhoea and 73% of these deaths are concentrated in 15 developing countries [1]. It is the second commonest cause of death due to infectious diseases in children under five and also a major cause of adult death in such countries [2]. The occurrence of diarrhoeal diseases also reflects the economic status of the country and has a complex effect on the country's economy by reducing the health of its work force. Three clinical syndromes of diarrhoea have been defined, namely acute watery diarrhoea, dysentery and persistent diarrhoea, each reflecting a different pathogenesis and requiring different approaches to treatment. Along with different bacteria and viruses, parasites are also important causes of diarrhoea in developing countries. However, parasitic infections are sometimes overlooked, leading after a period of time to an uncertain aetiology. In Kolkata, unpublished hospital-based data on the detection of diarrhoeal morbidity indicate that the incidence is huge. However, there have been no recent epidemiological surveys or systematic studies of occurrence and prevalence on which an estimate of the parasitic burden among different diarrheogenic cases could be based. A steady flow of diarrhoeal cases at the Infectious Disease Hospital, where there is a systematic clinical setup and a well-equipped parasitology laboratory is available at the National Institute of Cholera and Enteric Diseases (NICED), has helped to expand the scope of hospital surveillance for parasitic diarrhoea and enabled the present study to be undertaken. In this paper we describe the occurrence of three most abundant enteric parasites, *Giardia lamblia, Entamoeba histolytica *and *Cryptosporidium parvum*, in Kolkata and surrounding areas.

## Methods

Our target population comprised patients from different parts of Kolkata admitted to the Infectious Diseases (I. D.) Hospital between November 2007 and October 2008 with diarrhoeal complaints. Non-diarrhoea cases were not included in this study. A total of 1103 samples were screened; they were enrolled throughout the year by systematic sampling by our surveillance system. An epidemiological survey of the study population was also conducted, including stratification by age and sex, nature of parasitic infestations, month-wise prevalence of different parasites and GIS mapping of the areas where parasites are prevalent. The aim of this systematic study was to reveal the current burden of common enteric parasitic infestations in and around Kolkata.

### Study design

A laboratory-based surveillance system was introduced. The I. D. Hospital in Kolkata provides treatment to about 20,000 to 25,000 acute diarrhoea patients annually. Patients admitted to the hospital with diarrhoeal complaints were included in the study using a systematic sampling process: on two randomly selected days each week, every fifth patient with diarrhoea or dysentery but no associated complaints was enrolled. The system remained unbiased for sex and age of the patient at the time of selection. Faecal samples from all the enrolled patients were collected and analysed. A total of 19,500 patients were admitted to the I.D. Hospital during our study period; 1107 (5.6%) of these were enrolled by the system and 1103 fecal samples were processed for the study. The parasites were detected firstly by conventional microscopy, which is still considered the gold standard for any epidemiological study, but as this method has comparatively low sensitivity and has limitations in distinguishing among different parasites (e.g. *Entamoeba dispar *from *Emtamoeba histolytica*), we also used molecular detection techniques, ELISA and PCR, to increase the sensitivity and specificity of detection.

### Sample preparation

For microscopic analysis the samples were concentrated using the method of Ridley [3], mixed with 2.5% potassium dichromate for preservation [4] and stored at 4°C. Three separate aliquots from each sample were stored with no preservative at -80°C pending ELISA and PCR studies.

### Microscopic screening

Immediately they were received, the unpreserved samples were examined by microscopy. Three separate techniques were used to identify the parasites in the faecal samples: iodine wet mount staining for trophozoites and parasite cysts; modified Kinyoun's Acid fast staining for *Cryptosporidium sp*. [5]; and Trichrome staining for *Giardia *sp. and *Entamoeba *sp. [6].

### Molecular Screening

Antigen-capture Enzyme Linked Immunosorbent Assay (ELISA) was performed on all the samples to detect the above-mentioned parasites using commercially available kits, i.e. GIARDIA II, E. HISTOLYTICA II and CRYPTOSPORIDIUM II (TECHLAB, USA). Parasite DNA was isolated using a Stool DNA Isolation Kit (QIAGEN) according to the manufacturer's protocol, then PCR was performed on all samples using the following parasite-specific primers:-

#### (a) Giardia lamblia

A beta-giardin gene fragment-specific primer (MAH433F 5'-CATAACGACGCCATCGCGGCTCTCAGGAA-3' as forward primer and MAH592R 5'-TTTGTGAGCGCTTCTGTCGTGGCAGCGCTAA-3' as reverse primer) with a PCR amplicon size of 218 bp was used [7].

#### (b) Entamoeba histolytica

The target for PCR amplification was SSU rRNA (EH1 5'-GTACAAAATGGCCAATTCATTCAATG-3' as forward primer and EH2 5'-ACTACCAACTGATTGATAGATCAG-3' as reverse primer); the amplicon size is 135 bp [8]. The primers used are highly specific for *Entamoeba histolytica *and differentiate it from the non-pathogenic *Entamoeba dispar*.

#### (c) Cryptosporidium sp

The primers used were those described by Xiao *et al*. (1999) with an amplicon size of 1325 bp (18 SF: 5'-TTCTAGAGCTAATACATGCG-3' as forward primer and 18 SR: 5'-CCCTAATCCTTCGAAACAGGA-3' as reverse primer); then nested PCR was performed with an amplicon size of 825 bp (5'-GAAGGGTTGTATTTATTAGATAAAG-3' as forward primer and 5'-AAGGAGTAAGGAACAACCTCCA-3' as reverse primer) [9].

### Statistical analysis

The data from the above identification procedures and the epidemiological survey were entered into a pre-designed pro forma in the SQL server with an inbuilt entry validation checking facilitated program. Data were randomly matched and checked to establish consistency and validity. SPSS.14.0 was used for statistical analysis. In this study, positive cases in the inferential age group were explored for parasites (*Giardia lamblia, Cryptosporidium *sp and *Entamoeba histolytica*) by Multinomial Logistic Regression (MLR) [10,11].

### GIS analysis

A GIS map was constructed using the Choropleth Mapping Analysis System [12] for all patients enrolled by the surveillance system in order to establish the catchment areas of the parasites by evaluating their spatial distributions in Kolkata.

## Results

### Giardia lamblia

**Figure 1:**
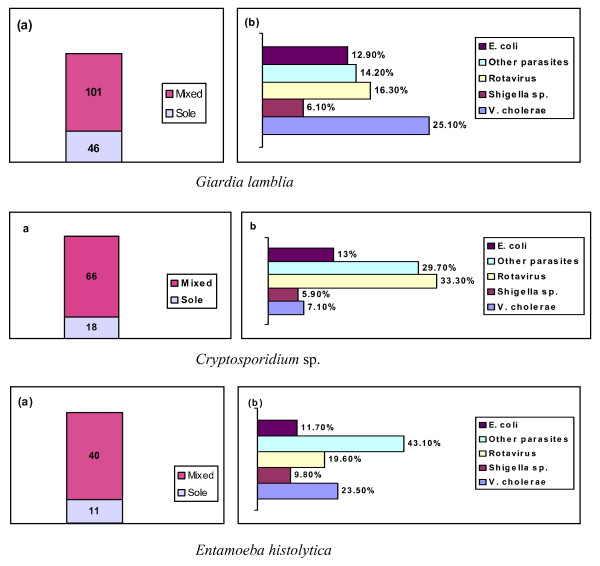
**Isolation status of *Giardia lamblia*, *Cryptosporidium sp*. and *Entamoeba histolytica***. Panel 1(a) Number of mixed and sole infection of *Giardia lamblia *in our study sample. Panel 1(b) Mixed infections of *Giardia lamblia *and other common pathogens showing a high degree of co-infection with *V. cholerae *followed by Rotavirus and other parasites. Panel 2(a) Number of mixed and sole infections with *Cryptosporidium *sp. in our study sample. Panel 2(b) Mixed infections of *Cryptosporidium *sp. with other common pathogens. The retuls show more co-infection with Rotavirus and other parasites than *V. cholerae*. Panel 3(a) Number of mixed and sole infections of *Entamoeba histolytica *in our study sample. Panel 3(b) Mixed infections of *Entamoeba histolytica *with other common pathogens. Like *Cryptosporidium*, *E. histolytica *showed most co-infection with the other parasites, followed by *V. cholerae *and Rotavirus.

Of the 1103 samples screened, 147 (13.3%) were positive for *Giardia lamblia *(Table 1). Among these, *Giardia lamblia *was the sole infection in 31.2% of cases and there was mixed infection with other common pathogens in the remainder. *Vibrio cholerae *and Rotavirus predominated in the mixed infections with *Giardia *(Fig. 1).

**Table 1:** Results of parasitic detection with three different techniques.

	*Giardia lamblia*	*Cryptosporidium *sp.	*Entamoeba histolytica*
MICROSCOPY	102	60	32

ELISA	147	84	51

PCR	147	84	51

**Figure 2:**
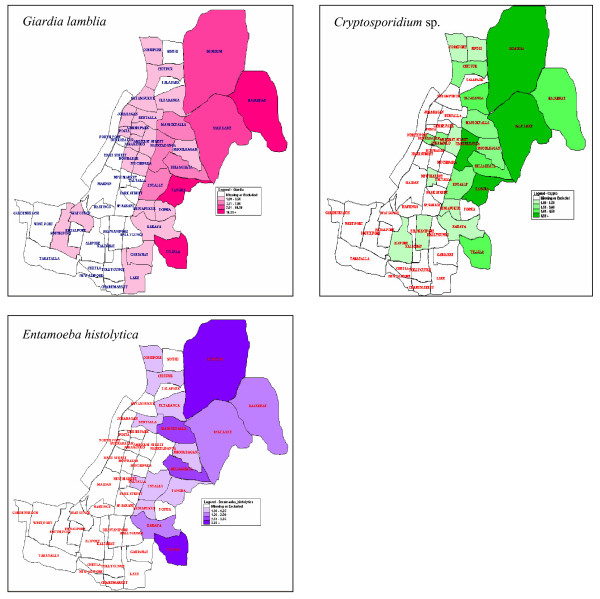
**Parasite prevalent areas**. Panel 1: GIS map of Kolkata showing the areas with high or low prevalence of *Giardia lamblia*. Areas such as Rajrhat, Tangra and Tiljala show the highest prevalence of *Giardia*. Saltlake, Dumdum, Beliaghata etc. also have high prevalences of *Giardia*. Panel 2: GIS map of Kolkata showing the areas with high or low prevalence of *Cryptosporidium *sp. The highest prevalence was seen in areas such as Salt Lake, Dumdum, Tangra etc. Panel 3: GIS map of Kolkata showing high prevalence of *Entamoeba histolytica *in areas such as Dumdum, Beliaghata, Maniktala etc.

GIS mapping of the patients admitted to the I.D. hospital indicated that the Rajarhat, Tangra and Tiljala areas of Kolkata had the highest incidence of Giardiasis, followed by Beliaghata and Salt Lake City (Fig. 2).

### Cryptosporidium sp

A total of 84 cases of *Cryptosporidium *sp. were diagnosed out of the 1103 samples screened (7.6% of the total surveillance population; Table 1). Among these,*Cryptosporidium *was the sole infection in 21.4% of cases and there was mixed infection with other common pathogens in the remainder; Rotavirus was predominant followed by other enteric parasites (Fig. 1). Bacterial infections mixed with *Cryptosporidium *sp. were less common than with *Giardia lamblia*.

The GIS map showed that Salt Lake City, Dumdum, Tangra and Narkeldanga had the highest incidences, followed by Rajarhat and Phoolbagan (Fig. 2).

### Entamoeba hisolytica

A total of 51 cases were diagnosed with *Entamoeba hisolytica *infection (4.6% of the total study population; Table 1). *Entamoeba hisolytica *was the sole infection in 21.5% of these cases and the remainder were mixed with other enteric parasites followed by viral and bacterial co-infections (Fig. 1).

The spatial distribution indicated that Dum Dum and Tiljala had the highest incidences of *Enatmoeba *infection followed by Narkeldanga and Phoolbagan (Fig. 2).

### Statistical Data

**Figure 3:**
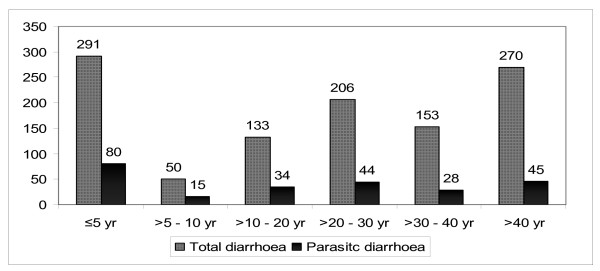
**Age-wise distridution of total and parasitic diarrhoea**. Comparison between total and parasitic diarrhoeal cases among six age groups in our study population.

Six age groups were assigned, *viz*. = 5 years, >5 – 10 years, >10 – 20 years, >20 – 30 years, >30 – 40 years and >40 years (Fig. 3). The = 5 year group showed the highest prevalence of parasitic infections. The extreme value of this group was fixed as the reference category and the other age groups were explored inferentially. The age group in which infections predominated was >5 – 10 years both for all parasites and for *Giardia *alone (Table 2).

**Table 2:** Multinomial Logistic Regression Models exploring age groups at significant risk for parasitic infestation

**Age**	**Pathogens**	**No. Of Positive**	**p-value**	**OR**	**95% CI**
≤ 5	Parasites	80	0.005*	1.79	1.193 – 2.687
	
	*G.lamblia*	46	0.020*	1.856	1.103 – 3.122

>5–10	Parasites	15	0.040*	2.043	1.033 – 4.039
	
	*G.lamblia*	14	<0.001*	3.937	1.862 – 8.326

>10–20	Parasites	34	0.059	1.62	0.983 – 2.672
	
	*G.lamblia*	25	0.009*	2.231	1.223 – 4.067

>20–30	Parasites	44	0.27	1.294	0.819 – 2.047
	
	*G.lamblia*	23	0.54	1.206	0.662 – 2.195

>30–40	Parasites	28	0.804	1.064	0.637 – 1.789
	
	*G.lamblia*	14	0.964	0.984	0.495 – 1.959

>40	Parasites	45	reference category		
				
	*G.lamblia*	25			

Total number of study population (N) = 1103, * = significant, OR = Odds Ratio, CI = Confidence Interval, Parasites = *Giardia lamblia*, *Entamoeba histolytica*, *Cryptosporidium *sp.

## Discussion

Infectious intestinal diseases are often self-limiting and in developing countries they are usually treated without proper diagnosis or information about aetiology. However, from the public health point of view, information about the pathogenic organism is very important for proper treatment, for monitoring trends, as an early warning for identifying outbreaks, and for introducing control measures and creating health policies.

**Figure 4:**
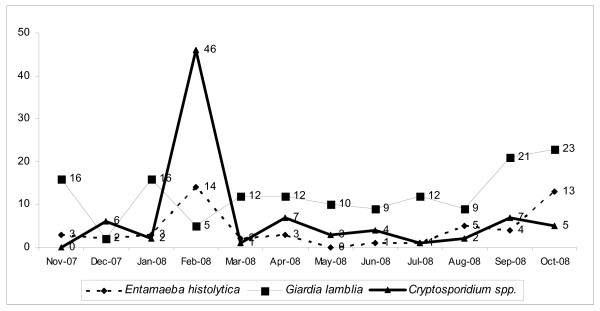
**Month-wise distribution of parasites detected**. Month-wise occurrence pattern of the three parasites. *Giardia lamblia *shows an almost steady curve ranging between 10 to 20 isolates per month, but *Cryptosporidium *sp. and *E. histolytica *show varying isolation status throughout the year.

In this study, the occurrence of *Giardia lamblia *remained almost unchanged throughout the year whereas the occurrence of *Cryptosporidium *sp. and *Entamoeba histolytica *showed differential seasonal distributions. For *E. histolytica *there was a specific trend towards increased occurrence in the post winter and post monsoon months (i.e. February to April and August to October) (Fig. 4). These sudden fluctuations could be the result of transient regional outbreaks caused by issues such as water sanitation; this needs further detailed investigation.

Importantly, parasitic infections mostly occurred in children and adults up to 20 years old. In our study population, the >5–10 year group showed a significantly high risk for *Giardia *infection and for other parasites. The most striking observation was the significant association of *Giardia *infection with adult patients up to 20 years old, although the = 5 year group was at the highest risk of parasitic infection in general. We also investigated the economic status of the study population; most of them came from a low socio-economic group.

Comparison between our detection rate and that obtained previously in the same area with a similar group of patients (unpublished data) indicates that the pattern of infection (aetiology) might be tending to change over time. Rates of infection with *Giardia *and *Cryptosporidium *have shown an increasing trend, but rates of infection with *Entamoeba histolytica *and its non-pathogenic asymptomatic variant *E. dispar *have tended to decrease. This is a striking observation and is potentially significant for considerations drug sensitivity and other environmental issues, which might be relevant to this slow but obvious change in the infection pattern.

## Conclusion

This study was performed to determine the prevalence of parasites causing diarrhoea in Kolkata and surrounding areas. Although no report on the surveillance of diarrhoeagenic parasitic aetiologies in this area has previously been published, we have found some interesting points that need to be highlighted. It is still unknown whether these parasites are the major causes of diarrhoea in the study location, but the results showing infection with each of these parasites show that they are potential causes of severe diarrhoea. The mixed aetiology results indicate that they may act as carriers for other pathogens, causing more severe diarrhoea.

As observed from the GIS map, parasitic infestation is most prevalent in the Tangra, Tiljala and Rajarhat areas. These are mostly slums. Also, most of our study population came from a low socio-economic group. Therefore, the most probable cause of infection may be water- and food-borne contamination. The study was conducted with an unbiased general population and no immunocompromised individuals were reported as such. However, the high occurrence of opportunistic parasitic infections (*Cryptosporidium *sp.) suggests poor hygiene and sanitation as well as malnutrition in the study population. Another striking observation was the significant association of parasitic diarrhoea, especially *Giardia *infection, with adults up to 20 years of age as well as children. From the month-wise distributions, we can assume a specific pattern of occurrence for all the parasites. Although *Enatmoeba *and *Cryptosporidium *showed some sporadic occurrence, *Giardia *showed a consistent infection rate throughout the year, from which we can infer that *Giardia *is endemic in Kolkata and surrounding areas.

This study provides important information about the prevalence of three most important intestinal parasites in this region. Continuation of this surveillance study is necessary to obtain an accurate understanding of the burden and cause of parasitic diarrhoea in this area.

## Competing interests

The authors declare that they have no competing interests.

## Authors' contributions

AKM (research fellow) performed the laboratory work and wrote the preliminary draft of the manuscript. MKB helped in the collection of samples and gathering background information from the study population. MG helped in the sample collection and surveillance. KR performed the statistical analysis of the data. SG designed the study, provided substantial intellectual contributions and supervision of the laboratory work and surveillance data collection, analyzed the data thoroughly for publication, drafted and revised the manuscript critically and gave the final approval of the version to be published.
